# Equalizing access to pandemic influenza vaccines through optimal allocation to public health distribution points

**DOI:** 10.1371/journal.pone.0182720

**Published:** 2017-08-30

**Authors:** Hsin-Chan Huang, Bismark Singh, David P. Morton, Gregory P. Johnson, Bruce Clements, Lauren Ancel Meyers

**Affiliations:** 1 Operations Research and Industrial Engineering, The University of Texas at Austin, Austin, TX, United States of America; 2 Industrial Engineering and Management Sciences, Northwestern University, Evanston, IL, United States of America; 3 Texas Advanced Computing Center, The University of Texas at Austin, Austin, TX, United States of America; 4 Health Emergency Preparedness and Response Section, Texas Department of State Health Services, Austin, TX, United States of America; 5 Integrative Biology, The University of Texas at Austin, Austin, TX, United States of America; 6 Santa Fe Institute, Santa Fe, New Mexico, United States of America; Pfizer Inc, UNITED STATES

## Abstract

Vaccines are arguably the most important means of pandemic influenza mitigation. However, as during the 2009 H1N1 pandemic, mass immunization with an effective vaccine may not begin until a pandemic is well underway. In the U.S., state-level public health agencies are responsible for quickly and fairly allocating vaccines as they become available to populations prioritized to receive vaccines. Allocation decisions can be ethically and logistically complex, given several vaccine types in limited and uncertain supply and given competing priority groups with distinct risk profiles and vaccine acceptabilities. We introduce a model for optimizing statewide allocation of multiple vaccine types to multiple priority groups, maximizing equal access. We assume a large fraction of available vaccines are distributed to healthcare providers based on their requests, and then optimize county-level allocation of the remaining doses to achieve equity. We have applied the model to the state of Texas, and incorporated it in a Web-based decision-support tool for the Texas Department of State Health Services (DSHS). Based on vaccine quantities delivered to registered healthcare providers in response to their requests during the 2009 H1N1 pandemic, we find that a relatively small cache of discretionary doses (DSHS reserved 6.8% in 2009) suffices to achieve equity across all counties in Texas.

## Introduction

Influenza pandemics—worldwide epidemics of novel influenza viruses—have occurred several times since the beginning of the twentieth century, each causing significant morbidity and mortality. The 2009 H1N1 influenza pandemic caused an estimated burden of 60.8 million cases, 274,000 hospitalizations, and 12,500 deaths in the U.S. [1]. Public health agencies around the globe are vigilantly preparing for pandemics and surveilling human, agricultural, and wildlife populations for threatening viruses. Two avian influenza viruses, H5N1 and H7N9, cause severe disease in humans but do not yet spread easily between humans [2, 3]. The U.S. Department of Health and Human Services (HHS) estimates that U.S. influenza deaths could reach 209,000 in a moderate pandemic and 1.9 million in a severe pandemic [4]. U.S. preparations for mitigating future pandemics include stockpiling tens of millions of courses of antiviral medications [5, 6], streamlining mass production of vaccines, and developing policies for prioritizing medical countermeasures and triggering non-pharmaceutical interventions, including school closures [4, 7].

The development and deployment of an effective pandemic vaccine involves several time-consuming steps, which can extend over six months [8–10]. The new influenza virus must be identified, adapted in public health laboratories for use in vaccine manufacturing, and optimized for vaccine production. Vaccines must then be produced and packaged in bulk, tested for safety and efficacy, distributed worldwide, injected into patients, and ultimately elicit immunological protection [8, 11]. As during the 2009 H1N1 pandemic, vaccines are likely to become available in batches, when demand greatly exceeds supply [12, 13]. Thus, strategic allocation of limited pandemic vaccines to prioritized groups and regions is critical, and has received significant attention. Dynamic prioritization strategies have been developed that target populations (e.g., school children, adults in specific age ranges, and people with certain health risks) or geographic regions to reduce infections, deaths, years of life lost, or economic cost [14–18]. Here, we introduce a model that takes such prioritized groups and regions as input and addresses the subsequent challenge of distributing vaccine supplies accordingly. Specifically, we optimally allocate a small reserve of discretionary doses to reach priority populations equitably across regions, while accounting for previously and concurrently distributed doses and the suitability of each vaccine type for each priority population.

During the 2009 H1N1 pandemic, the World Health Organization (WHO) provided donated vaccines to countries that could not afford commercially available vaccines. The WHO sought to provide equitable access across countries, and considered epidemiological and programmatic criteria [19]. The U.S. Centers for Disease Control and Prevention (CDC) allocated 2009 H1N1 vaccines to U.S. states pro rata (i.e., proportional to population) [20] consistent with HHS and U.S. Department of Homeland Security (DHS) pandemic recommendations for equitable vaccine distribution among and within states [21]. In the U.S. there were four types of vaccines that varied in acceptability for different populations [22]. Given the limited initial vaccine supply, the CDC prioritized populations according to guiding principles from its Advisory Committee on Immunization Practices (ACIP) [23] and expanded the target populations when vaccine supply became widely available [24].

Pro rata vaccination within states requires a representative geographic distribution of providers trained and available to immunize patients as vaccines become available. In 2009 many states distributed vaccines through voluntary healthcare providers in urban areas and further set up points of dispensing (PODs) to improve coverage for rural areas and other under-served populations [12, 25]. Moreover, following the 2009 H1N1 pandemic it was recommended that pharmacies may be most effective in reaching insured populations while state public health agencies should work to reach uninsured and low-income priority groups [20]. In logistical terms, our approach considers a *pull-based* distribution to healthcare providers based on their requests and a *push-based* distribution of quantities reserved for equalizing coverage [26, 27]. In future pandemics, state agencies will likely face such geographic challenges coupled with the complex task of apportioning multiple types of vaccines with variable supply, efficacy, and acceptabilities to achieve equitable coverage across multiple priority groups and regions.

We present an optimization model for allocating multiple types of vaccines to counties and priority groups within a state during an influenza pandemic. The model allows the state to provide the majority of doses to healthcare providers based on their requests, and then optimally allocates a small discretionary reserve of doses to achieve equal access across priority groups and geographic regions. Priority groups can be treated equally or weighted to reflect precedence. By “equal access” we mean *proportionally fair coverage*: a priority group in one county should have the same access to vaccines as the same priority group in another county. Furthermore, different priority groups should have relative levels of access corresponding to their policy-based precedence. Often, multiple possible allocations provide comparably high levels of coverage. Therefore, we perform a secondary optimization to effectively “break ties” among such proportionally fair allocations—or more precisely, allocations that achieve 99.9% of a proportionally fair allocation—to find one that achieves both policy simplicity and geographic equity. This produces allocations that reach priority groups fairly, while reducing the number of different vaccine types allocated to each priority group (policy simplicity), and providing similar proportions of each vaccine type across regions (geographic equity).

The ideas of access and equity in health and healthcare have been studied extensively; see, e.g., [28–32]. Clear, measurable definitions are critical to ensuring that scarce resources are allocated fairly across groups with different levels of social advantage [31]. We define equal access to mean: (i) for a specific priority group, the same number of vaccine doses should be available per priority group member across counties, and (ii) across priority groups, the number of doses per priority group member should be proportional to that group’s need, as specified by a policy-based weight that indicates that group’s precedence. These two notions of equal access are consistent with established concepts of horizontal and vertical equity [33, 34], respectively. However, our definition does not consider the greater mobility of advantaged social groups (that may allow them to cross county lines to receive vaccines) or other socioeconomic factors that may result in nonuniform uptake. With appropriate estimates, such factors could be incorporated in our model by forming additional location-priority group pairs and modifying precedence accordingly.

We demonstrate our approach using, as a case study, the 2009 H1N1 pandemic vaccination campaign in Texas. For each priority group and each county, we estimate the coverage that was achieved by doses distributed through registered healthcare providers and local health departments. We then apply our optimization framework to determine fair, simple, and equitable county-level allocations of the 6.8% of doses reserved by Texas in 2009 for POD-based distribution. We also perform a sensitivity analysis to determine the size of the discretionary reserve required to achieve proportional fairness in under-served priority populations. While our Web-based decision-support tool is tailored to the state of Texas, other jurisdictions may benefit from a similar approach. So, in addition to providing a retrospective analysis of 2009’s vaccination effort in Texas, we provide details that would allow development of an analogous tool, including how to populate it with requisite data.

## Methods

**Fig 1:**
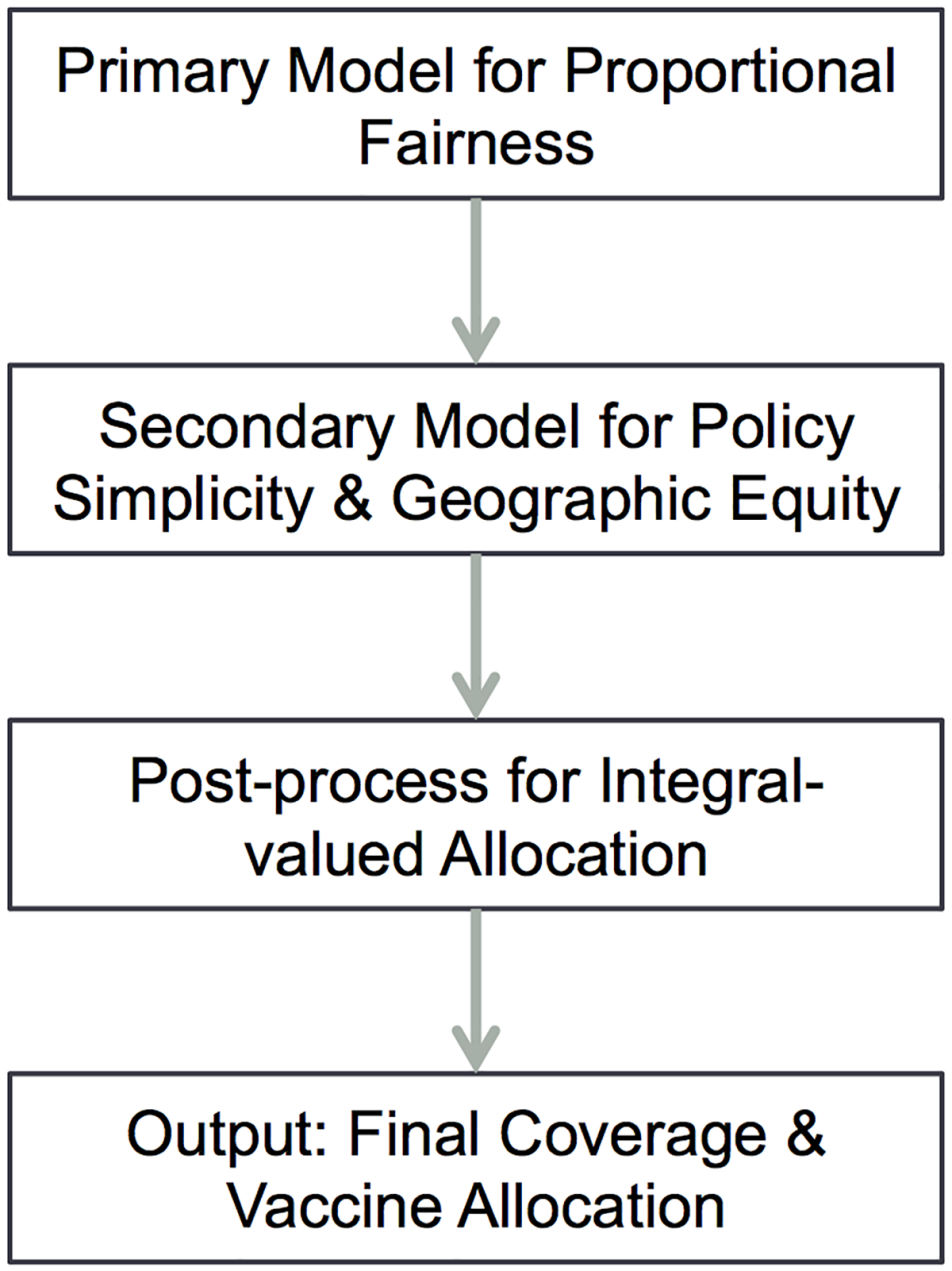
Overview of methods for allocating vaccines of multiple types to priority groups at multiple locations, maximizing proportional fairness with consideration of policy simplicity and geographic equity. The primary optimization model seeks proportionally fair coverage and the secondary model accounts for policy simplicity and geographic equity, while ensuring near optimality for proportional fairness. The post-processing step ensures integer-valued doses are allocated and then outputs the resulting final coverage and allocation.

Our approach consists of three stages (Fig 1). First, we build an optimization model (primary model) that seeks proportionally fair coverage for each location-priority group pair, where a location is a county in our analysis. Next, another optimization model (secondary model) takes as input the optimal coverage levels from the primary model and a sub-optimality tolerance for these levels; in our computation we allow at most 0.1% degradation in coverage. The secondary model aims to maximize policy simplicity and geographic equity while ensuring the gap between the optimal coverage and the resulting coverage is within the pre-defined tolerance for each location-priority group pair. Here, geographic equity is defined with respect to the eight health service regions of Texas as depicted in S1 Fig [35]. Lastly, we post-process the allocation from the secondary model to obtain integer-valued allocations. Our modeling framework aims for equitable allocation of multiple vaccine types to multiple location-priority group pairs, accounting for doses already allocated. Below we describe our optimization models and post-processing, but we reserve the mathematical details of the models for S1 Appendix.

### Primary model for proportional fairness

The objective of the primary optimization model is proportional fairness, i.e., each location-priority group pair should have equal coverage, when weighted by relative importance. We assign weights to each location-priority group pair to acknowledge their precedence, where the smallest weight is one. When weights differ by location-priority group pair, the model maximizes proportional fairness accordingly. For example, if one location-priority group pair has twice the weight of another, then the model aims for twice the coverage for the more highly weighted pair. This can indeed be achieved if: (i) both location-priority group pairs have coverage rates, after discretionary allocation, less than one, and (ii) allocations to the two pairs have at least one vaccine type in common in an optimal solution. The inputs include (i) coverage to date, weight, and population for each location-priority group pair, (ii) vaccine doses of different types available for discretionary allocation, and (iii) vaccine suitability rules. The model then provides as output the optimal coverage rate for each location-priority group pair.

### Secondary model for policy simplicity and geographic equity

The objectives of the secondary optimization model are sparsity of vaccine type-priority group allocations (policy simplicity) and similarity of allocations across regions (geographic equity). Since a priority group may be suitable for multiple types of vaccines, the primary model may have multiple optimal allocations, i.e., allocations with the same optimal coverage. While proportionally fair coverage is the primary objective, we prefer to reduce the number of vaccine types allocated to a priority group because this provides clearer direction to healthcare providers on which type of vaccine should be given to whom. In addition, we favor allocations with similar proportions of vaccine types across different geographic regions to reduce perceived geographic inequities. To give some flexibility in achieving the secondary objectives, we allow the coverage obtained from the secondary model to degrade slightly (at most 0.1% in the results we report) relative to the optimal coverage from the primary model. The inputs to the secondary model include (i) the optimal coverage rates of the primary model, (ii) a sub-optimality tolerance for these coverage rates, and (iii) all the inputs to the primary model. The model then provides as output the resulting coverage rate for each location-priority group pair and the associated allocation.

### Post-processing for integer-valued allocations

By using the primary and secondary models, we allocate available discretionary doses to location-priority group pairs in a proportionally fair manner with two secondary objectives. However, these two models ignore integrality of vaccine doses, resulting in fractional vaccine-dose allocations. Hence, we post-process to find a near-optimal solution that allocates integer-valued discretionary doses.

## Data for 2009 H1N1 pandemic case study

### Priority groups

In 2009 ACIP recommended the following groups be vaccinated with higher priority: (i) pregnant women, (ii) household contacts and caregivers for children younger than six months, (iii) healthcare and emergency medical services (EMS) personnel, (iv) people aged six months through 24 years, and (v) people aged 25 through 64 years at high risk for influenza-related complications [23]. Based on availability of demographic data, and assuming that the state will provide healthcare and EMS personnel with vaccines, we consider the following priority groups: (i) 0-3 year olds, (ii) 4-24 year olds, (iii) 25-64 year olds at high risk, (iv) pregnant women, and (v) infant caregivers. We assume all five groups have equal priority. S2 Appendix details how we estimated the size of each priority group population, with estimates largely based on demographic data from the 2010 U.S. Census, using county-level age distributions at one-year increments. In 2009, four vaccine types were used: (i) pre-filled syringe for baby (PFS baby), (ii) pre-filled syringe (PFS), (iii) multi-dose vial (MDV), and (iv) live attenuated influenza vaccine (LAIV) [22], with acceptabilities for the five priority groups shown in Table 1.

**Table 1:** Acceptability of 2009 H1N1 vaccine types for each priority group.

Acceptability	PFS baby	PFS	MDV	LAIV
0-3 years	1	0	0	0
4-24 years	0	1	1	1
25-64 years (high risk)	0	1	1	0
Pregnant women	0	1	1	0
Infant caregivers	0	1	1	1

1 indicates a vaccine type is acceptable for a priority group and 0 indicates it is not.

### Pull-based and push-based vaccine allocations

During the 2009 H1N1 pandemic, the Texas Department of State Health Services (DSHS) distributed vaccines using registered healthcare providers (RPs), local health departments (LHDs), and eight regional DSHS offices (HSRs), where the first two channels were pull-based (that is, allocated proportional to RP and LHD requests) and the third was push-based. We obtained the number of doses delivered to RPs in each county in Texas from [36] and to each LHD and HSR from [37, 38], but these data are aggregated over all vaccine types. We obtained the number of each vaccine type distributed on a weekly basis, aggregated statewide, from [39]. As of January 29, 2010 the percentages of PFS baby, PFS, MDV, and LAIV vaccines distributed were 3%, 17%, 60%, and 20%, respectively. To estimate the number of doses of each vaccine type distributed to county-priority group pairs by RPs and LHDs, we assumed each allocation reflected the statewide proportions, and assumed within a county, priority groups had access to acceptable vaccines in proportion to their population size.

## Results

### 2009 H1N1 pandemic vaccine allocation in Texas

In Texas, DSHS distributed the 2009 H1N1 pandemic vaccines received from the CDC to RPs, LHDs, and eight regional DSHS offices (HSRs) that serve 189 counties falling outside of LHDs. Large counties tend to have LHDs, with 151 of the 172 rural counties in Texas served by HSRs (S1 Fig). Of the 8.68 million vaccines received as of August 3, 2010, 76.9% were allocated to RPs and 16.3% were allocated to LHDs roughly in proportion to their requests (pull-based distribution), and the remaining 6.8% were distributed by DSHS through HSRs to boost coverage in counties with insufficient RP coverage (push-based distribution) [36–38]. The 189 counties in Texas served by HSRs had approximately 2.01 million individuals in our five priority groups and received approximately 1.23 million doses through RPs and HSRs. Under the simplifying assumption that each vaccine type is acceptable by each priority group, a perfectly proportionally fair allocation of Texas’ 6.8% discretionary reserve, coupled with RP allocations, would have achieved an *ideal coverage* of 61.1% of the priority population in the 189 counties served by HSRs.

**Fig 2:**
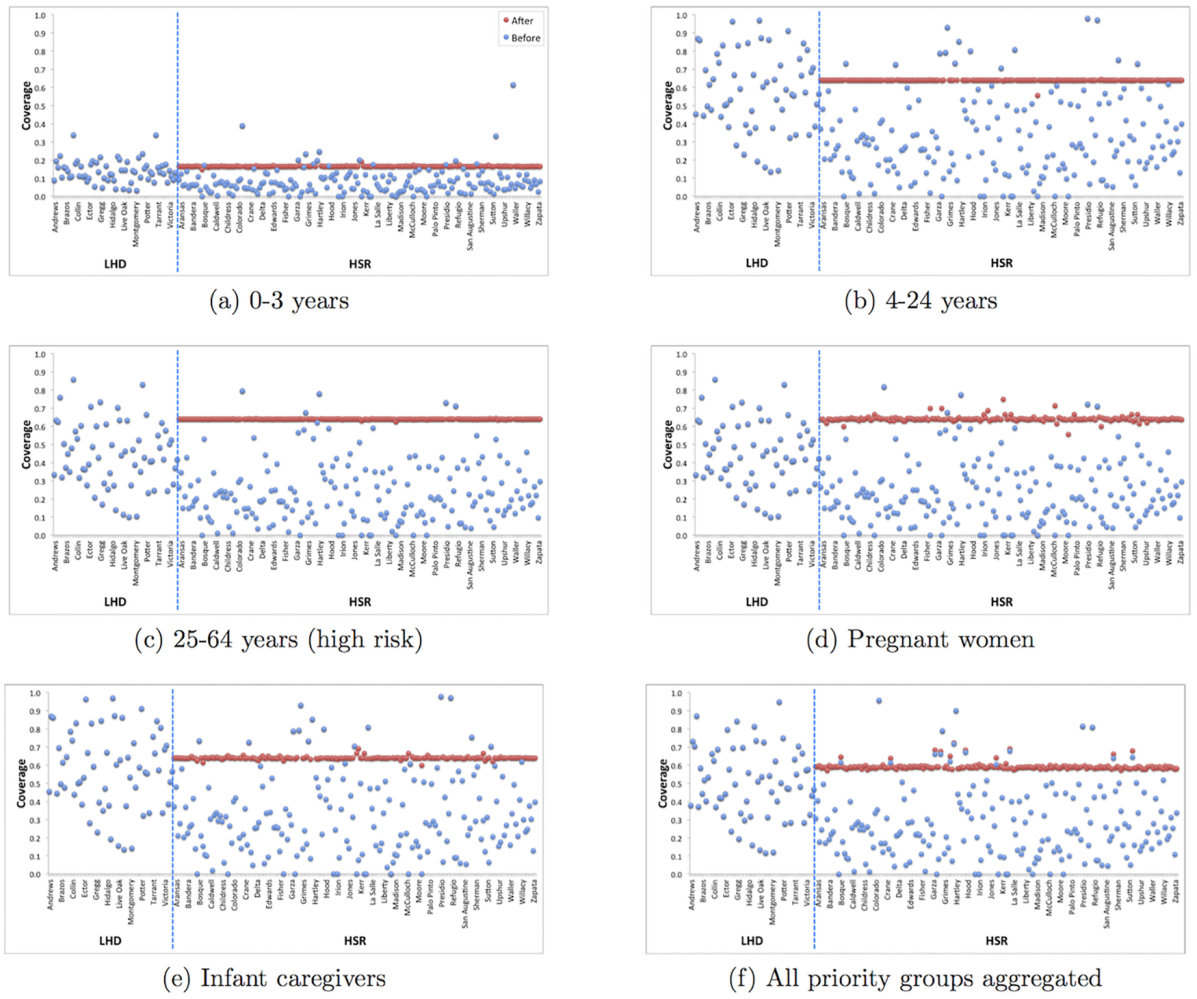
County-level vaccine coverage before (blue) and after (red) allocation of the 6.8% discretionary reserve, for each of the five priority groups and the aggregate prioritized populations. The left-hand side of each subfigure shows vaccine coverage for the 65 counties served by LHDs, which are not eligible for discretionary allocations; the right-hand side shows vaccine coverage for the other 189 counties that can receive discretionary doses. From left to right, the *x*-axis includes all 65 LHD counties and then 189 HSR counties in alphabetic order, but only displays a subset of the county names.

Henceforth, we focus on the 189 Texas counties served by HSRs. We estimated the coverage achieved by RPs and determined county-level allocations to HSRs that achieve proportionally fair coverage (Fig 2). The coverage of each priority group achieved through RPs and LHDs varies widely across counties (Fig 2, blue). Optimized allocation of the 6.8% of doses reserved for dispensing through HSR PODs brings most under-served counties up to a proportionally fair level (Fig 2, red). However, depending on vaccine acceptability, proportionally fair coverage ranged from 17% for 0-3 year olds (only 3% of doses were acceptable) to 64% in the other priority groups. Counties served by LHDs are not eligible for discretionary doses, and so inequalities stemming from disproportionate allocations via RPs and LHDs persist (Fig 2, left-hand side of each subfigure). Furthermore, some counties have populations so small that the discrete nature of doses changes coverage significantly; e.g., Loving County has nine individuals in the largest priority group of 4-24 year olds.

We performed a secondary optimization, selecting among proportionally fair allocations to favor both policy simplicity and geographic equity. The two allocations summarized in Tables 2 and 3 achieve the same level of proportional fairness to three significant digits, but the second solution is optimized for policy simplicity and geographic equity (Tables 2(b) and 3(b)). A proportionally fair allocation lacks simplicity if a given vaccine type is spread across all acceptable priority groups rather than narrowly focused on just one or two groups (Table 2(a)). Our secondary optimization finds an allocation with the fewest possible vaccine types allocated to each group (Table 2(b)). Note that 4-24 year olds receive three vaccine types in the solution of part (a) but only two in part (b); pregnant women reduce from two vaccine types in part (a) to one vaccine type in part (b); and, infant caregivers reduce from three vaccine types to one. These all represent improvements with respect to policy simplicity, which facilitates simpler messaging to vaccine providers and increases the likelihood that vaccination practices will be similar across regions. Moreover, a proportionally fair allocation is regarded less equitable geographically if the vaccine type proportions vary widely across regions (Table 3(a)). The secondary optimization favors similar proportions of each vaccine type across regions (Table 3(b)). This helps reduce the possibility of unfairness due to geographic differences in allocation.

**Table 2:** Percentage of doses allocated to each priority group by vaccine type for two proportionally fair allocations.

	(a) Prior to secondary optimization				(b) After secondary optimization			
Percentage (%)	PFS baby	PFS	MDV	LAIV	PFS baby	PFS	MDV	LAIV
0-3 years	100	0	0	0	100	0	0	0
4-24 years	0	12.6	55.7	31.7	0	0	73.3	26.7
25-64 years (high risk)	0	22.8	77.2	0	0	45.2	54.8	0
Pregnant women	0	44.2	55.8	0	0	100	0	0
Infant caregivers	0	28.2	37.2	34.6	0	0	0	100

The table shows the results for two proportionally fair allocations, (a) one prior to secondary optimization and (b) another further optimized to minimize the number of vaccine types assigned to each priority group and to homogenize the allocations across the eight HSRs.

**Table 3:** Percentage of doses allocated to each HSR by vaccine type for two proportionally fair allocations.

	(a) Prior to secondary optimization				(b) After secondary optimization			
Percentage (%)	PFS baby	PFS	MDV	LAIV	PFS baby	PFS	MDV	LAIV
HSR 1	3.5	26.3	46.2	24.0	3.5	14.5	57.2	24.8
HSR 2/3	2.8	12.8	66.3	18.1	2.8	18.1	60.4	18.7
HSR 4/5N	2.8	18.9	57.2	21.1	2.8	17.5	62.7	17.0
HSR 6/5S	3.0	13.8	62.2	21.0	3.0	14.1	64.3	18.6
HSR 7	2.9	16.6	60.2	20.3	2.9	16.1	55.1	25.9
HSR 8	3.1	16.8	61.1	19.0	3.1	19.0	63.2	14.7
HSR 9/10	3.3	31.1	44.2	21.4	3.3	19.2	54.2	23.3
HSR 11	4.3	21.3	52.9	21.5	4.3	13.2	59.9	22.6
Standard deviation	0.5	5.9	7.4	1.7	0.5	2.2	3.5	3.7
Range	1.5	18.3	22.1	5.9	1.5	6.0	10.1	11.2

The table shows the results for two proportionally fair allocations, (a) one prior to secondary optimization and (b) another further optimized to minimize the number of vaccine types assigned to each priority group and to homogenize the allocations across the eight HSRs.

### Discretionary reserve

The level of proportionally fair coverage attainable depends on the size of the discretionary reserve. A larger reserve provides more flexibility for equalizing access, but requires withholding doses from other (LHD-served) counties. Just for the moment, we make the simplifying assumption that all vaccine types are acceptable for all priority groups, and assess the trade-off between the *ideal coverage* of the focal counties and that of the remaining counties (Table 4). As the size of the discretionary reserve grows, the coverage increases quickly in the 189 counties receiving allocations, yet only decreases slightly in the other 65 counties.

**Table 4:** Maximum level of proportionally fair coverage attainable (% of the priority population) as a function of Texas’ discretionary reserve size.

Discretionary reserve (%)	Coverage in 189 HSR counties (%)	Coverage in 65 LHD counties (%)
1	38.1 (27.5, 2.8)	68.9 (52.4, 5.5)
3	46.0 (40.9, 1.5)	67.5 (51.4, 5.3)
5	54.0 (51.2, 0.9)	66.1 (50.3, 5.1)
**6.8**	**61.1 (59.3, 0.6)**	**64.9 (49.3, 4.9)**
9	69.9 (68.7, 0.3)	63.4 (48.2, 4.7)
11	77.8 (77.1, 0.2)	62.0 (47.1, 4.5)
13	85.8 (85.3, 0.1)	60.6 (46.1, 4.3)

The non-parenthetic results are under the assumption that all vaccine types are acceptable for all priority groups. The 189 HSR counties qualify for discretionary allocations, while the remaining 65 counties are served by LHDs. The 2009 H1N1 discretionary reserve was 6.8% of all doses (highlighted in bold). The parenthetical values are the medians and standard deviations in estimated coverage attainable (%) across counties when we instead assume the more restrictive priority group-vaccine type suitabilities from the 2009 H1N1 pandemic (Table 1). For the 65 LHD counties, these values are estimated directly from actual RP and LHD allocations in 2009; for the 189 HSR counties, these reflect coverage attained following optimization for proportional fairness with the specified discretionary reserve.

We now relax the universal suitability assumption and constrain the optimization using the priority group-vaccine type suitabilities from Table 1, and find that the “actual” coverage attainable is lower across all counties (Table 4, parenthetical values). Three factors contribute to this reduction. First, prior to allocation of the discretionary reserve, vaccines are apportioned to RPs and LHDs based on their requests, which do not perfectly match priority population sizes. This is the primary driver of reduced coverage in the LHD counties, and it affects the HSR counties when the discretionary reserve is small. Limited vaccine acceptability across priority groups also reduces the coverage attainable across the state (Table 4, parenthetical values), with larger discretionary reserve ameliorating the impact in HSR counties. Finally, the discrete nature of allocations can produce mismatches between the desired coverage and vaccine supply, especially in counties with small populations. The variation in coverage attainable is smaller across the HSR counties than the LHD counties (Table 4, parenthetical standard deviations), reflecting the evenness achieved via proportionally fair optimization. The size of the 2009 H1N1 discretionary reserve (6.8%) provides the most balanced *ideal coverage*. However, when accounting for vaccine-type suitabilities the smaller discretionary reserve of 5% provides the most balanced coverage from the table. Still smaller reserves fail to make up shortfalls in RP-based allocations, and larger reserves shortchange regions that rely exclusively on pull-based allocations.

## Discussion

During future influenza pandemics, public health agencies will be charged with making rapid vaccine allocation decisions to effectively and ethically protect geographically, demographically, and medically diverse populations. This challenge is compounded by the dynamic and uncertain nature of both vaccine supplies and the epidemic itself. Our optimization framework was motivated by a retrospective assessment of the 2009 vaccine allocation process in Texas, and aims to streamline future decision making. As demonstrated for the 2009 H1N1 pandemic in Texas, the method identifies geographic allocations of multiple vaccine types that provide proportionally fair coverage across any number of priority groups with any combination of vaccine acceptabilities. It considers the geographic distribution of the priority group populations and accounts for coverage from prior or concurrent vaccination efforts, while allowing specific groups and locations to be prioritized over others. Through a secondary analysis, the method finds fair allocations that also achieve policy simplicity and geographic equity.

We implemented this approach in a user-friendly application for the Texas DSHS and regional offices in the state’s eight HSRs [40]. The decision-maker specifies: (i) the HSRs or counties to receive allocations, (ii) vaccine priority groups (pre-populated options include pregnant women, infants, infant caregivers, first responders, high-risk conditions, and customizable age ranges), (iii) any number of vaccine types, along with their acceptability for each priority group and number of available doses, and (iv) estimates of current vaccine coverage for each priority group in each county (either as number of vaccine doses or proportion vaccinated). The tool optimizes sequentially for proportional fairness, followed by policy simplicity and geographic equity, and displays the recommended allocations through interactive graphs, maps, and tables. Given census data on priority group populations and the four inputs listed above, this approach can be applied elsewhere and at any scale to assist in bringing under-served populations up to a proportionally fair level of vaccination coverage, using a discretionary cache of doses.

In our case study of Texas’ 2009 H1N1 pandemic vaccine allocation process, four types of vaccines were distributed to five priority groups via three channels: registered healthcare providers (RPs), Local Health Departments (LHDs) serving one or more counties, and health service regions (HSRs). The first two channels received pull-based allocations roughly in proportion to requests they submitted to DSHS, while the last channel provided a push-based conduit to reach under-served populations in the 189 counties in Texas without LHDs. In 2009, Texas DSHS reserved 6.8% of all H1N1 vaccine doses allocated to the state for HSR distribution. We analyzed reserves ranging from 1% to 13% of the state allocation. We found that a 6.8% reserve more than sufficient for achieving proportionally fair coverage in the target HSR counties, and found that the smaller reserve of 5% strikes the best balance between provisioning RPs and LHDs and serving the populations they miss. Our optimal allocation achieves policy simplicity by specifying a single vaccine type for three of the five priority groups and two vaccine types for the remaining two groups, and achieves geographic equity by sending each HSR roughly the same (proportional) distribution across vaccine types (see Tables 2(b) and 3(b)).

The extent to which optimization can shrink coverage gaps and achieve proportional fairness depends on the size of the discretionary reserve and the acceptability of priority groups for available vaccine types. If the reserve is too small or supplies of vaccine types are imbalanced, then proportional fairness may not be attainable geographically or among priority groups. In practice, Texas’ 2009 distribution was relatively decentralized; once a regional office (HSR) received discretionary vaccine doses from DSHS, it had autonomy regarding subsequent allocations. Our results assume both that the state follows the recommended regional allocation and that the regional offices follow the recommended county-level allocations. The Web-based decision-support tool is designed for direct use by both state and regional offices, and to facilitate communication of the optimized allocations.

Although the optimization models we solved aspire to achieve equal assess across all priority groups in the HSR counties, the method can differentially weight priority groups, locations, or combinations, if desired. If one population is assigned twice the weight of another, then the model aims for a two-to-one ratio in coverage between the two target populations. The method readily extends from a single allocation decision to a time-dynamic, adaptive decision process that accounts for prior allocations and changing coverage targets. This may be critical if the epidemiological situation and our understanding of risk factors vary geographically or evolve during the pandemic.

As we have discussed, in 2009 Texas considered a centralized push-based allocation only to HSR counties, whose priority group population is about 15% of the state-wide total. Without our optimization framework, a simple calculation suggests an ideal coverage rate of 64.3% (total number of doses divided by total priority group population). Under the “HSR-only policy” that we analyze, we instead attain roughly 50% coverage with a 5% discretionary reserve (see Table 4), although some non-HSR counties are well short of this value and others exceed it (see Fig 2). We now apply our same optimization framework under the policy that the discretionary reserve can be allocated to all counties, rather than just HSR counties. Under a 5% discretionary reserve, we obtain 44.6% aggregate coverage (analog of parenthetic median value in Table 4 and “red line” for the aggregate prioritized population in Fig 2). Under larger discretionary reserves of 15%, 25%, and 35% the respective coverage values grow to 58.4%, 63.2%, and 64.2%. Here, larger discretionary reserves are required to achieve overall equity because non-HSR counties contain about 85% of the priority group population in Texas.

More broadly, our approach can determine a fair allocation of any critical resource with complicated combinations of target populations and resource suitability, such as age-group or risk-group specific vaccines, medications, or life support equipment. However, some important factors are not yet incorporated, including different costs and uptake rates associated with specific distribution channels and elimination of overlap in priority group categories. Our case study assumes that all doses will reach their target populations with equal probability and cost, regardless of location, vaccine type, and vaccine provider. However, the match between providers and patients, based on factors such as insurance, provider specialization, and patient priority group, will likely vary spatially, and hence thwart uniform uptake. When distribution resources or adherence within priority groups vary, there may be critical trade-offs. There is overlap between the priority groups of infant caregivers and pregnant women as well as between these two groups and 4-24 year olds and 25-64 year olds at high risk, but our analysis ignores the associated double counting. It was unknown how many doses were required to achieve immunity when ACIP guidelines were released [20]. Clinical testing subsequently revealed that one dose sufficed for most people, but children 3-8 years old required two doses, and those younger than three required two half-doses. Our analysis does not account for this, and our notion of coverage is based on one dose per person.

## Supporting information

S1 Fig The eight health service regions in the state of Texas. (TIFF)

S1 Appendix Optimization modeling framework. (PDF)

S2 Appendix Priority group population estimation. (PDF)

## References

[1] Shrestha SS, Swerdlow DL, Borse RH, Prabhu VS, Finelli L, Atkins CY, et al Estimating the Burden of 2009 Pandemic Influenza A (H1N1) in the United States (April 2009-April 2010). Clinical Infectious Diseases. 2011; 52(Suppl 1): S75–S82. 10.1093/cid/ciq012 21342903

[2] Centers for Disease Control and Prevention (CDC). Highly Pathogenic Avian Influenza A (H5N1) in People. 2015. Available: http://www.cdc.gov/flu/avianflu/h5n1-people.htm. Accessed 30 July 2016.

[3] Centers for Disease Control and Prevention (CDC). Avian Influenza A (H7N9) Virus. 2014. Available: http://www.cdc.gov/flu/avianflu/h7n9-virus.htm. Accessed 30 July 2016.

[4] U.S. Department of Health and Human Services (HHS). HHS Pandemic Influenza Plan. 2005. Available: http://www.flu.gov/planning-preparedness/federal/hhspandemicinfluenzaplan.pdf. Accessed 30 July 2016.

[5] Centers for Disease Control and Prevention (CDC). Strategic National Stockpile (SNS). 2016. Available: http://www.cdc.gov/phpr/stockpile/stockpile.htm. Accessed 30 July 2016.

[6] U.S. Department of Health and Human Services (HHS). Considerations for Antiviral Drug Stockpiling by Employers in Preparation for an Influenza Pandemic. 2005. Available: http://www.flu.gov/planning-preparedness/business/antiviral_employer.pdf. Accessed 30 July 2016.

[7] Homeland Security Council. National Strategy for Pandemic Influenza. 2005. Available: http://www.flu.gov/planning-preparedness/federal/pandemic-influenza-strategy-2005.pdf. Accessed 30 July 2016.

[8] World Health Organization (WHO). Pandemic Influenza Vaccine Manufacturing Process and Timeline. 2009. Available: http://www.who.int/csr/disease/swineflu/notes/h1n1_vaccine_20090806/en/. Accessed 30 July 2016.

[9] Haaheim LR, Madhun AS, Cox R. Pandemic Influenza Vaccines—The Challenges. Viruses. 2009; 1(3): 1089–1109. 10.3390/v1031089 21994584 PMC3185517

[10] Rebmann T, Zelicoff A. Vaccination against Influenza: Role and Limitations in Pandemic Intervention Plans. Expert Review of Vaccines. 2012; 11(8): 1009–1019. 10.1586/erv.12.63 23002981

[11] Centers for Disease Control and Prevention (CDC). How Influenza (Flu) Vaccines Are Made. 2015. Available: http://www.cdc.gov/flu/protect/vaccine/how-fluvaccine-made.htm. Accessed 30 July 2016.

[12] Texas Department of State Health Services (DSHS). Final After Action Report: DSHS Response to the Novel H1N1 Pandemic Influenza Event. 2010. Available: http://www.cidrap.umn.edu/sites/default/files/public/php/566/566_aar.pdf. Accessed 30 July 2016.

[13] Centers for Disease Control and Prevention (CDC). 2009 H1N1 Vaccine Doses Allocated, Ordered, and Shipped by Project Area. 2010. Available: http://www.cdc.gov/h1n1flu/vaccination/vaccinesupply.htm. Accessed 30 July 2016.

[14] Medlock J, Galvani AP. Optimizing Influenza Vaccine Distribution. Science. 2009; 325(5948): 1705–1708. 10.1126/science.1175570 19696313

[15] Keeling MJ, White PJ. Targeting Vaccination against Novel Infections: Risk, Age and Spatial Structure for Pandemic Influenza in Great Britain. Journal of the Royal Society Interface. 2010; 8(58): 661–670. 10.1098/rsif.2010.0474 PMC3061093 20943682

[16] Wu JT, Riley S, Leung GM. Spatial Consideration for the Allocation of Pre-Pandemic Influenza Vaccination in the United States. Proceedings of the Royal Society B: Biological Sciences. 2007; 274(1627): 2811–2817. 10.1098/rspb.2007.0893 17785273 PMC2288690

[17] Araz OM, Galvani A, Meyers LA. Geographic Prioritization of Distributing Pandemic Influenza Vaccines. Health Care Management Science. 2012; 15(3): 175–187. 10.1007/s10729-012-9199-6 22618029 PMC4295509

[18] Matrajt L, Halloran ME, Longini IM Jr. Optimal Vaccine Allocation for the Early Mitigation of Pandemic Influenza. PLoS Computational Biology. 2013; 9(3): e1002964 10.1371/journal.pcbi.1002964 23555207 PMC3605056

[19] World Health Organization (WHO). Report of the WHO Pandemic Influenza A(H1N1) Vaccine Deployment Initiative. 2012. Available: http://www.who.int/influenza_vaccines_plan/resources/h1n1_deployment_report.pdf. Accessed 30 July 2016.

[20] Stroud C, Nadig L, Altevogt BM. The 2009 H1N1 Influenza Vaccination Campaign: Summary of a Workshop Series. D.C.: The National Academies Press; 2010. 21595118

[21] U.S. Department of Health and Human Services (HHS) and U.S. Department of Homeland Security (DHS). Guidance on Allocation and Targeting Pandemic Influenza Vaccine. 2014. Available: http://www.flu.gov/images/reports/pi_vaccine_allocation_guidance.pdf. Accessed 30 July 2016.

[22] Centers for Disease Control and Prevention (CDC). 2009 H1N1 Monovalent Influenza Vaccine Dosage, Administration, and Storage. 2009. Available: http://www.cdc.gov/h1n1flu/vaccination/dosage.htm. Accessed 30 July 2016.

[23] Centers for Disease Control and Prevention (CDC). 2009 H1N1 Vaccination Recommendations. 2009. Available: http://www.cdc.gov/h1n1flu/vaccination/acip.htm. Accessed 30 July 2016.

[24] Centers for Disease Control and Prevention (CDC). 2009 H1N1: Overview of a Pandemic. 2010. Available: http://www.cdc.gov/h1n1flu/yearinreview/yir7.htm. Accessed 30 July 2016.

[25] Rambhia KJ, Watson M, Sell TK, Waldhorn R, Toner E. Mass Vaccination for the 2009 H1N1 Pandemic: Approaches, Challenges, and Recommendations. Biosecur Bioterror. 2010; 8(4): 321–330. 10.1089/bsp.2010.0043 21043791

[26] Hopp WJ, Spearman ML. Factory Physics, 3rd ed New York: McGraw-Hill; 2008.

[27] Simchi-Levi D, Kaminsky P, Simchi-Levi E. Designing and Managing the Supply Chain: Concepts, Strategies, and Cases. New York: McGraw-Hill; 2000.

[28] Joseph AE, Phillips D. Accessibility and Utilization—Geographical Perspectives on Healthcare Delivery. New York: Harper & Row; 1984.

[29] Penchansky R, Thomas JW. The Concept of Access: Definition and Relationship to Consumer Satisfaction. Medical Care. 1981; 19(2): 127–140. 7206846 10.1097/00005650-198102000-00001

[30] Aday LA, Andersen RM. Equity of Access to Medical Care: A Conceptual and Empirical Overview. Medical Care. 1981; 19(12 Suppl): 4–27. 10.1097/00005650-198112001-00004 7339313

[31] Braveman P, Gruskin S. Defining Equity in Health. Journal of Epidemiology and Community Health. 2003; 57(4): 254–258. 10.1136/jech.57.4.254 12646539 PMC1732430

[32] Bayoumi AM. Equity and Health Services. Journal of Epidemiology and Community Health. 2009; 30(2): 176–182. 10.1057/jphp.2009.9 19597449

[33] Culyer AJ. Equity—Some Theory and Its Policy Implications. Journal of Medical Ethics. 2001; 27(4): 275–283. 10.1136/jme.27.4.275 11479360 PMC1733434

[34] Mooney G. Vertical Equity in Health Care Resource Allocation. Health Care Analysis. 2000; 8(3): 203–215. 10.1023/A:1009439917796 11186022

[35] Texas Department of State Health Services (DSHS). The Health Service Regions. 2014. Available: https://www.dshs.state.tx.us/regions/state.shtm. Accessed on 30 July 2016.

[36] Texas Department of State Health Services (DSHS). H1N1 Vaccine Doses by County to Texas Registered Providers. 2010. Available: http://www.dshs.state.tx.us/txflu/H1N1-Doses-Providers.pdf. Accessed 30 July 2016.

[37] Texas Department of State Health Services (DSHS). H1N1 Vaccine Doses to Texas Local Health Departments. 2010. Available: http://www.dshs.state.tx.us/txflu/H1N1-Doses-LHD.pdf. Accessed 30 July 2016.

[38] Texas Department of State Health Services (DSHS). H1N1 Vaccine Doses to Texas DSHS Regional Offices. 2010. Available: http://www.dshs.state.tx.us/txflu/H1N1-Doses-Regions.pdf. Accessed 30 July 2016.

[39] Texas Department of State Health Services (DSHS). Internal Dashboard Report for H1N1 Vaccine. 2010.

[40] Texas Department of State Health Services (DSHS) and The University of Texas at Austin. Texas Pandemic Flu Toolkit. 2013. Available: http://flu.tacc.utexas.edu. Accessed 30 July 2016.

