# Supplementary figures and images for: Equalizing access to pandemic influenza vaccines through optimal allocation to public health distribution points
Source: PLoS One. 2017 Aug 30;12(8):e0182720. doi: 10.1371/journal.pone.0182720 (PMC5576642; doi:10.1371/journal.pone.0182720)

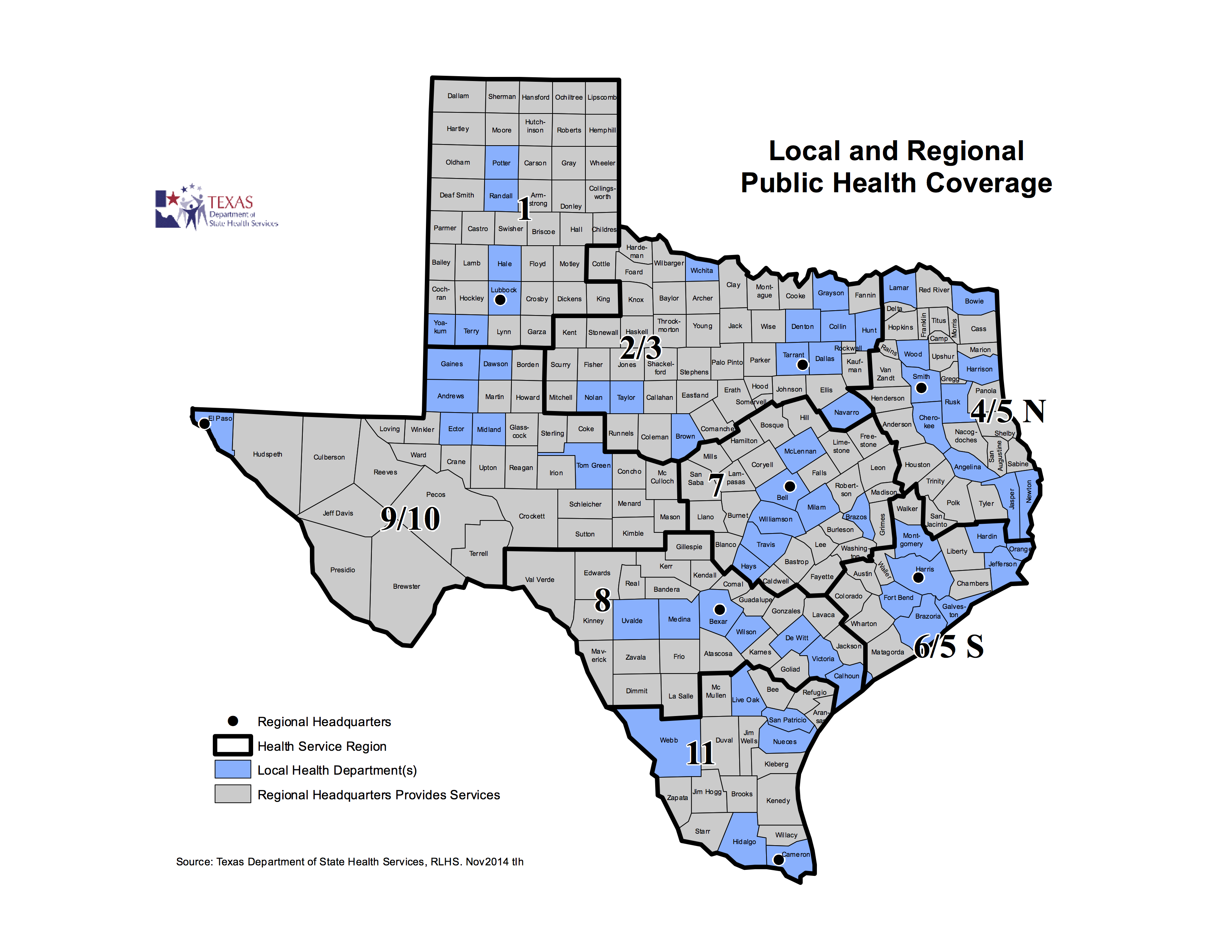

Supplement: S1 Fig — (TIFF) [file pone.0182720.s001.tiff]
