# Supplementary material for: Equalizing access to pandemic influenza vaccines through optimal allocation to public health distribution points
Source: PLoS One. 2017 Aug 30;12(8):e0182720. doi: 10.1371/journal.pone.0182720 (PMC5576642; doi:10.1371/journal.pone.0182720)
Supplement: S1 Appendix — (PDF) [file pone.0182720.s002.pdf]

S1 Appendix: Optimization modeling framework

We formulate two optimization models (primary and secondary) and employ a post-processing step, which together allocate available discretionary doses to locations and, in turn, to prespecified priority groups, seeking proportional fairness with policy simplicity and geographic equity in mind. The objective of the primary model is proportional fairness while the objectives of the secondary model are policy simplicity and geographic equity. We describe the two models and the post-processing step in detail below.

Primary optimization model

We index locations (counties in our analysis) by $i \in I$, priority groups by $j \in J$, and vaccine types by $k \in K$. The set K_j denotes vaccine types acceptable for priority group j, L denotes location-priority group pairs (i, j), and G denotes location, priority-group, vaccine-type triples, i.e., all (i, j, k) such that $k \in K_j$ and $(i, j) \in L$. Prior coverage of priority group j in location i is given by $f_{ij} = (\sum_{k \in K_j} m_{ijk})/n_{ij}$, where m_{ijk} is the number of doses of vaccine type k previously allocated to location-priority group pair (i, j), and n_{ij} is the population size of location-priority group pair (i, j). If $f_{ij} \geq 1$ then we exclude (i, j) from the eligibility set L. Finally, b_k denotes the number of doses of vaccine type k available for discretionary allocation, and w_{ij} denotes the importance weight, i.e., precedence, assigned to location-priority group pair (i, j).

The decision variables, Q_{ijk}, allocate discretionary doses of type k to location-priority group

pair (i, j). The optimization model is as follows:

$$\min_{Q} \quad \sum_{(i,j)\in L} w_{ij} n_{ij} \left[1 - \frac{1}{w_{ij}} \left(f_{ij} + \frac{\sum_{k\in K_j} Q_{ijk}}{n_{ij}}\right)\right]^2 \tag{1a}$$

$$\text{s.t.} \quad \sum_{(i,j):(i,j,k)\in G} Q_{ijk} \le b_k, \forall k \in K \tag{1b}$$

$$f_{ij} + \frac{\sum_{k\in K_j} Q_{ijk}}{n_{ij}} \le 1, \forall (i,j) \in L \tag{1c}$$

$$Q_{ijk} \ge 0, \forall (i,j,k) \in G. \tag{1d}$$

If all location-priority group pairs are equally important (i.e., all weights w_{ij} are set to one) then the objective function in (1a) is the sum of the squares of vaccine shortfalls,

$$1 - \left(f_{ij} + \frac{\sum_{k\in K_j} Q_{ijk}}{n_{ij}}\right),$$

weighted by the location-priority group population sizes across all such pairs. In the optimization model, we minimize this sum of population-weighted shortages. Below, we provide sufficient conditions under which model (1)'s optimal allocations maximize proportional fairness.

Constraint (1b) ensures that the allocated doses of vaccine type k do not exceed the available doses; constraint (1c) indicates that coverage cannot exceed one for any eligible location-priority group pair; and, constraint (1d) precludes negative allocations.

We add weights ($w_{ij} \ge 1$) for each location-priority group pair to acknowledge their relative importance, where the value of the least important location-priority group pair under consideration is one. When the weights differ, instead of seeking equal coverage for priority

groups in each location, we seek equal coverage when weighted by the proportionality constants, w_{ij}. So, if one location-priority group pair has twice the weight of another, we seek twice the coverage rate for the higher priority pair. As Theorem 1 shows, this can indeed be achieved for two location-priority group pairs under the following conditions: (i) both of their final coverage rates, after discretionary doses are allocated, are less than one, and (ii) these two pairs have at least one common type of available discretionary vaccines allocated in an optimal solution.

With the proper inputs, model (1) provides an optimal allocation, denoted Q^*_{ijk}, and, in turn, the optimal coverage rate of each pair, denoted g_{ij}, i.e.,

$$g_{ij} = f_{ij} + \frac{\sum_{k \in K_j} Q^*_{ijk}}{n_{ij}}. \tag{2}$$

Here, we show that the objective function in (1a), when combined with the constraints (1b)-(1d), seeks to provide an allocation of available discretionary doses in a manner of proportional fairness.

Theorem 1. *By using model (1) to allocate available discretionary vaccine doses, the optimal coverage rates of two location-priority group pairs, (i,j) and (i',j'), denoted g_{ij} and $g_{i'j'}$, are proportional to their weights, w_{ij} and $w_{i'j'}$, if (i) both g_{ij} and $g_{i'j'}$ as defined in equation (2) are less than 1; and, (ii) (i,j) and (i',j') have a common type of available discretionary vaccine allocated in an optimal solution.*

Proof. Let λ_k and ν_{ij} be the Lagrange multipliers of constraints (1b) and (1c), respectively. Let $F(Q)$ denote the objective function in (1a). Then, we have

$$\frac{\partial F}{\partial Q_{ijk}} = 2\left[-1 + \frac{1}{w_{ij}}\left(f_{ij} + \frac{\sum_{k \in K_j} Q_{ijk}}{n_{ij}}\right)\right].$$

As a result, the Karush-Kuhn-Tucker optimality conditions for the convex quadratic program (1) (see, e.g., [1]) are then:

Primal feasibility: constraints (1b), (1c), and (1d)

Dual feasibility:

$$\lambda_k^* + \frac{\nu_{ij}^*}{n_{ij}} \geq 2\left[1 - \frac{1}{w_{ij}}\left(f_{ij} + \frac{\sum_{k\in K_j} Q_{ijk}^*}{n_{ij}}\right)\right], \forall(i,j,k) \in G \tag{3a}$$

$$\lambda_k^* \geq 0, \forall k \in K \tag{3b}$$

$$\nu_{ij}^* \geq 0, \forall (i,j) \in L \tag{3c}$$

Complementary slackness:

$$\lambda_k^* \left(b_k - \sum_{(i,j):(i,j,k)\in IJK} Q_{ijk}^*\right) = 0, \forall k \in K \tag{4a}$$

$$\nu_{ij}^* \left[1 - \left(f_{ij} + \frac{\sum_{k\in K_j} Q_{ijk}^*}{n_{ij}}\right)\right] = 0, \forall (i,j) \in L \tag{4b}$$

$$Q_{ijk}^* \left\{\lambda_k^* + \frac{\nu_{ij}^*}{n_{ij}} - 2\left[1 - \frac{1}{w_{ij}}\left(f_{ij} + \frac{\sum_{k\in K_j} Q_{ijk}^*}{n_{ij}}\right)\right]\right\} = 0, \forall (i,j,k) \in G. \tag{4c}$$

Consider two (i,j) and (i',j') pairs that satisfy hypotheses (i) and (ii). By hypothesis (i) and complementary slackness condition (4b), we have $\nu_{ij}^* = \nu_{i'j'}^* = 0$. By hypothesis (ii), we have $Q_{ijk}^* > 0$ and $Q_{i'j'k}^* > 0$ for some k and hence by (4c) for that k:

$$\frac{2-\lambda_k^*}{2} = \frac{1}{w_{ij}}\left(f_{ij} + \frac{\sum_{k\in K_j} Q_{ijk}^*}{n_{ij}}\right) = \frac{1}{w_{i'j'}}\left(f_{i'j'} + \frac{\sum_{k\in K_j} Q_{i'j'k}^*}{n_{i'j'}}\right).$$

Using the definition of g_{ij} from equation (2), we have

$$\frac{g_{ij}}{g_{i'j'}} = \frac{w_{ij}}{w_{ij'}}.$$

□

Secondary optimization model

We index geographic health service regions by $h \in H$. We form three more index sets: I_h, O, and P, where I_h is the subset of counties in region h; O is the subset of priority group-vaccine type pairs ($O \subseteq J \times K$) for which priority group j is suitable for vaccine type k; and, P is the subset of region, priority group, vaccine type triples ($P \subseteq H \times J \times K$), which indicates priority group-vaccine type pairs in region h with (j, k) in set O.

We define decision variables V_{jk}, Y_{hjk}, and $\overline{Y}_{jk}$: V_{jk} is a binary variable, which takes value 1 if we allocate discretionary doses of type k to priority group j and takes value 0 otherwise; Y_{hjk} is the regional coverage of priority group j in region h from discretionary doses of type k; and, $\overline{Y}_{jk}$ is the average regional coverage of priority group j from discretionary doses of type k. Instead of requiring the optimal coverage levels from the primary model, we allow the final coverage of a location-priority group pair to be at most ε away from optimality to provide more opportunity to improve secondary objectives. In our analysis presented in the main text, we use $\varepsilon = 0.001$.

The secondary optimization model is as follows:

$$\min_{Q,V,Y,\overline{Y}} \quad \sum_{(j,k)\in O} V_{jk} + \sum_{(h,j,k)\in P} \frac{|Y_{hjk} - \overline{Y}_{jk}|}{|H|} \tag{5a}$$

$$\text{s.t.} \quad \sum_{(i,j):(i,j,k)\in G} Q_{ijk} \leq b_k, \forall k \in K \tag{5b}$$

$$f_{ij} + \frac{\sum_{k\in K_j} Q_{ijk}}{n_{ij}} \leq 1, \forall (i,j) \in L \tag{5c}$$

$$Q_{ijk} \geq 0, \forall (i,j,k) \in G \tag{5d}$$

$$f_{ij} + \frac{\sum_{k\in K_j} Q_{ijk}}{n_{ij}} \geq g_{ij}(1-\varepsilon), \forall (i,j) \in L \tag{5e}$$

$$\sum_{i\in I:(i,j,k)\in G} Q_{ijk} \leq M \cdot V_{jk}, \forall (j,k) \in O \tag{5f}$$

$$Y_{hjk} = \frac{\sum_{i\in I_h:(i,j,k)\in G} Q_{ijk}}{\sum_{i\in I_h:(i,j)\in L} n_{ij}}, \forall (h,j,k) \in P \tag{5g}$$

$$\overline{Y}_{jk} = \frac{\sum_{h \in H} Y_{hjk}}{|H|}, \forall (j,k) \in O \tag{5h}$$

$$V_{jk} \in \{0,1\}, \forall (j,k) \in O. \tag{5i}$$

The objective function in (5a) consists of two terms. The first term represents the number of vaccine type-priority group pairs that receive doses, and the second term measures the variation of vaccine types allocated across health service regions. In order to understand the second term, fix a (j,k) pair for the moment. As defined mathematically in constraints (5g) and (5h), Y_{hjk} is the regional coverage of priority group j in region h that comes from discretionary doses of vaccine type k, and $\overline{Y}_{jk}$ is its average across all health service regions. The average absolute deviation,

$$\sum_{h \in H} \frac{|Y_{hjk} - \overline{Y}_{jk}|}{|H|},$$

represents the dispersion vaccine type k contributes to the coverage of priority group j among regions. By summing this average absolute deviation across $j \in J$, we obtain the dispersion vaccine type k contributes to the coverage among regions. Lastly, we sum across $k \in K$ to form a measure of variation of vaccine types allocated among health service regions. We could assign different weights to the two terms in (5a) to adjust the relative importance of policy simplicity and geographic equity. However, we obtain desirable results, as we describe in the main text, with equal weights.

In addition to constraints (5b)-(5d), which replicate constraints from the primary model, we have five other sets of constraints to satisfy. Constraint (5e) ensures that the final coverage of a location-priority group pair is no more than ε away from the optimal proportionally fair coverage of the primary model. Constraints (5f) and (5i) calculate the number of vaccine type-priority group pairs that receive doses using the "big M" method, where M is a constant that is large enough so that constraint (5f) is vacuous if $V_{ik} = 1$. Constraints (5g) and (5h)

define Y_{hjk} and $\overline{Y}_{jk}$ for measuring the variation of vaccine types allocated among health service regions.

Model (5) takes as input the optimal proportionally fair coverage rates (g_{ij}) from the primary model. Then, model (5) provides an optimal allocation, denoted Q_{ijk}^{**}, and the associated coverage rate of each location-priority group pair, which considers proportional fairness and the secondary objectives.

Post-processing for integer-valued allocations

By using the primary and secondary models, we allocate available discretionary doses to eligible location-priority group pairs in a proportionally fair manner with two secondary objectives. However, these two models ignore integrality of vaccine doses. Hence, we construct a post-processing step to find a near-optimal solution that allocates integer-valued discretionary doses. First, for each vaccine type k, we round each Q_{ijk}^{**} from model (5) down to the nearest integer, denoted $\lfloor Q_{ijk}^{**} \rfloor$. Next, we calculate the difference between the sum of Q_{ijk}^{**} across all (i, j) pairs in subset L and that of $\lfloor Q_{ijk}^{**} \rfloor$. This difference represents extra doses from the fractional part of each Q_{ijk}^{**}. We sort eligible (i, j) pairs in descending order of their fractions and add one dose to each $\lfloor Q_{ijk}^{**} \rfloor$ by this order until all the extra doses are allocated.

Computation

The primary and secondary optimization models are solved using the commercial software modeling language GAMS [2], which calls the CPLEX [3] optimization solver. We can run the software to solve the models on a server at `http://flu.tacc.utexas.edu/` or on a laptop. On a 1.7 GHz Intel Core i7 MacBook Air using GAMS version 24.7.3 and CPLEX version 12.6.3, the three-step solution process takes about 4 seconds for a single value of the discretionary reserve (e.g., 6.8%). This computation time includes the software creating a

model instance from input data, solving the primary and secondary models, and performing the post-processing step.

References

[1] Boyd S, Vandenberghe L. Convex Optimization. Cambridge, UK: Cambridge University Press; 2004.

[2] GAMS—A User's Guide. 2017. Available: https://www.gams.com/latest/docs/userguides/GAMSUsersGuide.pdf. Accessed on 17 June 2017.

[3] CPLEX Optimizer, IBM. 2017. Available at: https://www-01.ibm.com/software/commerce/optimization/cplex-optimizer/. Accessed on 17 June 2017.
