# Supplementary material for: Equalizing access to pandemic influenza vaccines through optimal allocation to public health distribution points
Source: PLoS One. 2017 Aug 30;12(8):e0182720. doi: 10.1371/journal.pone.0182720 (PMC5576642; doi:10.1371/journal.pone.0182720)
Supplement: S2 Appendix — (PDF) [file pone.0182720.s003.pdf]

S2 Appendix: Priority group population estimation

As we mention in the main text, during the 2009 H1N1 influenza pandemic, the Advisory Committee on Immunization Practices (ACIP) from the Centers for Disease Control and Prevention (CDC) recommended the following groups be vaccinated with higher priority: (i) pregnant women, (ii) household contacts and caregivers for children younger than six months, (iii) healthcare and emergency medical services (EMS) personnel, (iv) people aged six months through 24 years, and (v) people aged 25 through 64 years at high risk for influenza-related complications [1]. Based on availability of demographic data, and the notion that healthcare and EMS personnel will receive vaccines separately from our distribution system, we instead consider the following five priority groups: (i) 0-3 year olds, (ii) 4-24 year olds, (iii) 25-64 year olds at high risk, (iv) pregnant women, and (v) infant caregivers. We describe how we can obtain estimates for the population of each of these priority groups for counties in Texas, as opposed to providing the specific values for the priority groups during the 2009 H1N1 pandemic.

0-3 year olds

Population counts for children under the age of 3 years are available from the Texas State Data Center [2] for the year 2010 for all counties in Texas.

4-24 year olds

Population counts for 4-24 year olds are again available from the Texas State Data Center [2] for the year 2010 for all counties in Texas.

25-64 year olds at high risk

Once again, population counts for 25-64 year olds are available from the Texas State Data Center [2] for the year 2010 for all counties in Texas. We obtain the fraction of the population

in this age group at high risk from the CDC [3] and Medlock et al. [4]. The high-risk category includes individuals deemed to have a high risk for having influenza complications because they have a chronic condition, have an immunocompromised condition, or are pregnant. The proportion of the population in the high-risk category varies by age. Chronic conditions for adults are: "current asthma, chronic bronchitis, emphysema, coronary heart disease, angina, heart attack, diabetes, stroke, weak kidney, epilepsy, cerebral palsy, movement disorders, and muscular dystrophies" [3, 4].

Pregnant women

We use the following notation in estimating the population of pregnant women:

Indicies and sets

$i \in I$	counties in Texas	
$j \in J$	age groups by year $\{10, 11, \ldots, 50\}$	
$k \in K$	age group bins for available data:	for births: $\{10, 15, 18, 20, 30, 40\}$
		for fetal losses: $\{10, 15, 18, 20, 25, 30, 35, 40\}$
		for abortions: $\{10, 15, 20, 25, 30, 35, 40\}$

Data

w_{ij}	count of women in age group j in county i
B_{ik}	cumulative live births from women of age bin k in county i
A_{ik}	cumulative abortions from women of age bin k in county i
F_k	cumulative fetal losses from women of age bin k per 1,000 women
p_b	proportion of the year a woman is pregnant when she has a live birth: $\frac{9}{12}$
p_f	proportion of the year a woman is pregnant when she has a fetal loss: $\frac{2}{12}$
p_a	proportion of the year a woman is pregnant when she has an abortion: $\frac{3}{12}$

Estimated parameters

b_{ij} live births from women of age group j in county i

f_j fetal losses from women of age group j per 1,000 women

a_{ij} abortions from women of age group j in county i

Given b_{ij}, f_j, and a_{ij}, we estimate the number of pregnant women of age group j at any given time of the year for county i by:

$$p_b b_{ij} + p_a a_{ij} + p_f \frac{w_{ij}}{1000} f_j.$$

We seek b_{ij}, f_j, and a_{ij} at the yearly age resolution indexed by $j \in J$, but certain data are available only in coarser age bins, $k \in K$, as we indicate above. To obtain live births with yearly resolution, b_{ij}, we use a county-specific smoothed weighted interpolation scheme of B_{ik} (available using data from [5, 6, 7]), with weights arising from state-wide age-specific live birth information available from the CDC [5]. For abortions, a_{ij}, we used a county-specific cubic Hermite interpolation scheme making use of the available data, A_{ik}, from the Texas Department of State Health Services (DSHS) [8]. For fetal loss rates, f_j, we make use of CDC recommendations [9], (see also MacDorman and Kirmeyer [10] and Ventura et al. [11]), to use a national rate, thus removing geographic considerations. Again, we use a cubic Hermite interpolation scheme making use of fetal loss data, F_k, available from Ventura et al. [11] to obtain the required rate.

Infant caregivers

We restrict attention to parents for estimating the population of infant caregivers. Let sp_i denote the fraction of children living in single-parent households in county i, and let B_i denote the number of live births reported in county i. We estimate sp_i using a five-year average from 2006-2010 with the county-specific percentage of children living in families

headed by a parent without a spouse present in the home [12]. Estimates of B_i are available from DSHS [13].

Using one caregiver per household for a single-parent household and two caregivers otherwise, we estimate the number of infant caregivers by:

$$B_i sp_i + 2B_i(1 - sp_i).$$

The fraction of children with single-parent households, sp_i, differs widely, from 5% in McMullen County to 56.5% in San Saba County. Thus, we see the above approach as providing potentially important geographic resolution, and hence we do not perform a state-wide aggregation.

References

[1] Centers for Disease Control and Prevention (CDC). 2009 H1N1 Vaccination Recommendations. 2009. Available: `http://www.cdc.gov/h1n1flu/vaccination/acip.htm`. Accessed 30 July 2016.

[2] Texas State Data Center. Texas Population Estimates Program. 2015. Available: `http://txsdc.utsa.edu/Data/TPEPP/Estimates/Index.aspx`. Accessed 30 July 2016.

[3] Centers for Disease Control and Prevention (CDC). National Health Interview Survey, 2006-2008. 2016. Available: `http://www.cdc.gov/nchs/nhis.htm`. Accessed 30 July 2016.

[4] Medlock J, Meyers LA, Galvani AP. Supplemental Material for Optimizing Allocation for a Delayed Influenza Vaccination Campaign. 2009. Available: `http://www.bio.utexas.edu/research/meyers/_docs/publications/MedlockPLoSCurrentsSupplement09.pdf`. Accessed 30 July 2016.

[5] Centers for Disease Control and Prevention (CDC). National Vital Statistics Reports. 2016. Available: http://www.cdc.gov/nchs/products/nvsr.htm. Accessed 30 July 2016.

[6] March of Dimes. PeriStats. 2016. Available: http://www.marchofdimes.com/peristats. Accessed 30 July 2016.

[7] Texas Department of State Health Services (DSHS). Texas Health Data. 2015. Available: http://soupfin.tdh.state.tx.us/. Accessed 30 July 2016.

[8] Texas Department of State Health Services (DSHS). Induced Terminations of Pregnancy by Age and County of Residence Texas, 2010. 2012. Available: http://www.dshs.state.tx.us/chs/vstat/vs10/t34.shtm. Accessed 30 July 2016.

[9] Centers for Disease Control and Prevention (CDC). Estimating the Number of Pregnant Women in a Geographic Area. 2014. Available: http://www.cdc.gov/reproductivehealth/Emergency/PDFs/PregnacyEstimatoBrochure508.pdf. Accessed 30 July 2016.

[10] MacDorman MF, Kirmeyer S. The Challenge of Fetal Mortality. NCHS Data Brief. 2009; 16. Available: http://www.cdc.gov/nchs/data/databriefs/db16.pdf. Accessed 30 July 2016.

[11] Ventura SJ, Abma JC, Mosher WD, Henshaw SK. Estimated Pregnancy Rates by Outcome for the United States, 1990-2004. National Vital Statistics Reports. 2008; 56(15). Available: http://www.cdc.gov/nchs/data/nvsr/nvsr56/nvsr56_15.pdf. Accessed 30 July 2016.

[12] Kids Count Data Center. Children in Single-Parent Families 2006-2010. 2016. Available: http://datacenter.kidscount.org/data/tables/3059-children-in-single-parent-families?loc=45&loct=2#ranking/5/any/true/1000/any/8193. Accessed 30 July 2016.

[13] Texas Department of State Health Services (DSHS). Reported Pregnancies for Women Age 15-44. 2012. Available: http://www.dshs.state.tx.us/chs/vstat/vs10/t14a.shtm. Accessed 30 July 2016.
